# Rates and costs of invasive pneumococcal disease and pneumonia in persons with underlying medical conditions

**DOI:** 10.1186/s12913-016-1432-4

**Published:** 2016-05-13

**Authors:** Derek Weycker, Raymond A. Farkouh, David R. Strutton, John Edelsberg, Kimberly M. Shea, Stephen I. Pelton

**Affiliations:** Policy Analysis Inc. (PAI), Four Davis Court, Brookline, MA 02445 USA; Pfizer Inc., Collegeville, PA USA; Boston University Schools of Medicine and Public Health, Boston, MA USA; Boston Medical Center, Boston, MA USA

**Keywords:** *Streptococcus pneumoniae*, Pneumococcal infections, Pneumonia, Comorbidity, Cost and cost analysis

## Abstract

**Background:**

The presence of certain underlying medical conditions is known to increase the risk of pneumococcal disease in persons of all ages and across a wide spectrum of conditions, as demonstrated in two recent evaluations. Corresponding estimates of attributable economic costs have not been well characterized. We thus undertook a retrospective evaluation to estimate rates and costs of pneumococcal disease among children and adults with and without underlying medical conditions in the United States.

**Methods:**

Data were obtained from three independent healthcare claims repositories. The study population included all persons enrolled in participating health plans during 2007–2010, and was stratified into subgroups based on age and risk profile: healthy; at-risk, due to selected comorbid conditions; and high-risk, due to selected immunocompromising conditions. At-risk and high-risk conditions, as well as episodes of invasive pneumococcal disease (IPD) and all-cause pneumonia (PNE), were identified via diagnosis, procedure, and drug codes. Rates and healthcare costs of IPD and PNE (2010US$) among at-risk and high-risk persons were compared with those from age-stratified healthy counterparts using incidence rate ratios (IRR) and cost ratios.

**Results:**

Rates of IPD and PNE were consistently higher among at-risk persons (IRR = 4.1 [95 % CI 3.9–4.3] and 4.5 [4.49–4.53]) and high-risk persons (IRR = 10.3 [9.7–11.0] and 8.2 [8.2–8.3]) of all ages versus their healthy counterparts. Rates were notably high for at-risk persons with ≥2 conditions (IRR = 9.0 [8.4–9.7] and 10.3 [10.3–10.4]), as well as those with asthma (IRR = 3.4 [3.0–3.8] and 4.5 [4.47–4.53]) or diabetes (IRR = 4.3 [4.0–4.6] and 4.7 [4.6–4.7]). Healthcare costs totaled $21.7 million per 100,000 at-risk person-years and $58.5 million per 100,000 high-risk person-years, which were 8.7 [8.5–8.8] and 23.4 [22.9–23.8] times higher than corresponding costs for healthy persons.

**Conclusions:**

Rates and costs of IPD and PNE are substantially higher among persons with certain chronic and immunocompromising conditions versus those without any such conditions. Rates and costs for persons with asthma and diabetes were especially increased, and rates and costs for individuals with ≥2 at-risk conditions approached those among persons with high-risk conditions.

**Electronic supplementary material:**

The online version of this article (doi:10.1186/s12913-016-1432-4) contains supplementary material, which is available to authorized users.

## Background

*Streptococcus pneumoniae* (pneumococcus) has long been recognized as a major cause of serious but uncommon invasive diseases such as meningitis and bacteremic pneumonia, and much more common non-invasive diseases such as non-bacteremic pneumonia and acute otitis media. Not surprisingly then, the economic burden of pneumococcal disease in the United States (US) has been reported to be substantial. In one recent evaluation, Huang and colleagues estimated the direct cost of pneumococcal disease among persons of all ages in the US to have been $3.5 billion in 2004 [1]. In another evaluation, Weycker and colleagues estimated the direct cost of pneumococcal disease among older US adults (i.e., persons aged ≥ 50 years) to have been $3.7 billion in 2007 [2]. Finally, Wroe and colleagues projected that from 2004–2040, as the size of the US population grows (especially the elderly) and absent intervention, the number of hospitalizations for pneumococcal pneumonia will nearly double and the total economic burden of pneumococcal pneumonia will increase by $2.5 billion annually [3].

In the study by Weycker et al., the effect of patients’ underlying risk of pneumococcal disease on both the cost per case of disease and the overall economic burden of pneumococcal disease were examined, but only to a limited extent [2]. We therefore examined in greater detail, and for persons of all ages, the clinical and economic burden of pneumococcal disease in the US in relation to underlying risk status. Risk profiles were determined by the presence of selected underlying chronic illnesses that are currently listed in guidelines promulgated by the US Advisory Committee on Immunization Practices (ACIP) as indications for pneumococcal vaccination, as well as conditions that might increase the risk of infection based on limited data from other studies [4–7].

## Methods

### Study design

A retrospective cohort design was employed. Study cohorts were identified at the beginning of each calendar year of observation―from 2007 to 2010―and study subjects were stratified in terms of age and the presence of underlying medical conditions (i.e., risk profile) based on information recorded at any time prior to January 1^st^ of that calendar year. For each cohort, episodes of invasive pneumococcal disease (IPD) and all-cause pneumonia (PNE) were ascertained during the 1-year period beginning on January 1^st^ of each corresponding year and ending on December 31^st^ of that year (or date of loss to follow-up, if earlier). (All-cause PNE was selected as a study measure—rather than pneumococcal pneumonia—because pneumococcal pneumonia is under-reported in healthcare claims and ~30 % of all cause PNE is estimated to be due to *Streptococcus pneumonia*e [7, 8]. Stratum-specific rates of IPD and PNE (per 100,000 person-years) were estimated over the entire study period. Subjects who met criteria for inclusion in multiple calendar years contributed data to each cohort for which they were eligible.

### Data source

Data were obtained from three large integrated healthcare claims repositories and spanned January 1, 2006 through December 31, 2010. The three databases—Truven Health Analytics MarketScan® Commercial Claims and Encounters and Medicare Supplemental and Coordination of Benefits Databases, Intercontinental Marketing Services’ (IMS) LifeLink™ PharMetrics Health Plan Claims Database, and OPTUM Research Database—include medical (i.e., facility and professional-service) claims and outpatient pharmacy claims from private US health plans. Together, these three geographically diverse repositories capture healthcare claims information for >35 million plan members annually.

Data available from each facility and professional-service claim included dates and places of service, diagnoses (International Classification of Diseases, Ninth Revision, Clinical Modification [ICD-9-CM]), procedures performed/services rendered [ICD-9-CM, Healthcare Common Procedure Coding System (HCPCS)], and quantity of services (professional-service claims). Data available for each outpatient pharmacy claim included the drug dispensed, dispensing date, quantity dispensed, and number of days supplied. Medical and pharmacy claims also included amounts paid by health plans and patients for services rendered, or standardized estimates of the cost of services. In addition, selected demographic and eligibility information (including age/year of birth, sex, geographic region of residence, and dates of plan eligibility) were available.

The study databases were de-identified prior to their release to study investigators. The study databases have been evaluated and certified by independent third parties to be in compliance with the Health Insurance Portability and Accountability Act (HIPAA) of 1996 statistical de-identification standards and to satisfy the conditions set forth in Sections 164.514 (a)-(b)1ii of the HIPAA Privacy Rule regarding the determination and documentation of statistically de-identified data. Use of the study databases for health services research is therefore fully compliant with the HIPAA Privacy Rule and federal guidance on Public Welfare and the Protection of Human Subjects [9]. Permission to use the data sources for analyses described herein was requested by study investigators and was granted by the data vendors. Detailed descriptions of the three healthcare claims repositories are provided in the Additional file 1.

### Study population

The study population comprised all persons of all ages who were enrolled in participating health plans on the first day of one or more calendar years from 2007 to 2010. Study subjects were stratified based on their age (0–17, 18–64, and ≥65 years) and risk profile (“healthy”, “at-risk”, and “high-risk”) as of the beginning of each year. At-risk persons included those who were immunocompetent with ≥1 chronic medical condition. High-risk persons included those who were immunocompromised, immunosuppressed, or had a cochlear implant. At-risk and high-risk categories were mutually exclusive and thus, for example, persons considered immunosuppressed because of cancer were included in the high-risk category only, even if they also had an at-risk condition. Healthy persons included those without evidence of at-risk or high-risk conditions.

The lists of at-risk and high-risk conditions included all those set forth by the ACIP in its recommendations for pneumococcal vaccination; the at-risk list also included the following conditions: asthma, chronic steroid use, trisomy 21, prematurity/low birth weight, neuromuscular/seizures disorders, rheumatoid arthritis, systemic lupus erythematosis, and Crohn’s disease [10]. At-risk and high-risk conditions were ascertained using information (i.e., ICD-9-CM diagnosis codes, ICD-9-CM/HCPCS procedure codes, and drug codes from the HCPCS/National Drug Code [NDC] systems) recorded any time prior to the beginning of the study year. Persons with at-risk conditions were stratified by the number of conditions; those with asthma and diabetes were further stratified by disease severity. Operational algorithms that were employed to identify at-risk and high-risk conditions are set forth in Additional file 1: Tables S1 and S2.

Persons who were not continuously eligible for comprehensive health (i.e., medical and drug) benefits for at least 1 year prior to January 1^st^ of ≥1 corresponding years were excluded from the study population. Infants (i.e., those <12 months of age as of January 1^st^) in a given year, were not subject to this exclusionary criterion.

### Study measures

Study measures included episodes and associated healthcare costs of selected manifestations of pneumococcal infection, including IPD and PNE. All-cause pneumonia was chosen as an outcome measure in preference to pneumococcal pneumonia because, although *S. pneumoniae* infection is recognized as the most common cause of pneumonia, the diagnostic code for pneumococcal pneumonia appears uncommonly in healthcare claims data (due to the relatively small number of times *S. pneumoniae* is successfully cultured in clinical practice from patients with pneumonia).

Episodes of IPD and PNE were ascertained beginning on January 1^st^ and ending on December 31^st^ of each study year (or the date of loss to follow-up, if prior to December 31st). Disease episodes requiring inpatient care were identified using operational algorithms based on ICD-9-CM diagnosis codes, and spanned the duration of hospitalization. Episodes of IPD requiring outpatient care only were identified based on ICD-9-CM diagnosis codes and HCPCS/NDC codes for antibiotic therapy (±5 days), while episodes of PNE requiring outpatient care only were identified based on ICD-9-CM diagnosis codes, CPT codes for chest x-ray, and HCPCS/NDC codes for antibiotic therapy (±5 days); outpatient episodes spanned a maximum of 90 days (Additional file 1: Table S3). Multiple episodes during a given study year were included if they were separated by at least 90 days.

A disease-attributable approach was used to estimate healthcare costs whereby expenditures for disease-related services rendered during the episode of care were tallied. Disease-related services were identified based on diagnosis codes (principal diagnosis only on inpatient claims), procedure codes, and drug codes. The cost of inpatient episodes included hospitalizations, ambulatory visits, and pharmacotherapy during the episode; the cost of outpatient episodes included all outpatient visits and pharmacotherapy during the episode. Costs were estimated using healthcare expenditures reported on claims (i.e., amounts reimbursed to healthcare providers by health plans and patients for services rendered), and were expressed in 2010 US dollars.

### Analyses

Crude analyses were undertaken to estimate rates of disease episodes (per 100,000 person-years) within each age group by risk profile and individual risk condition, using data from all three study repositories. Differences in rates of disease—and corresponding 95 % confidence intervals (CIs)—between at-risk/high-risk persons and healthy counterparts were expressed as incidence rate ratios (IRR) using Poisson regression analysis.

Mean episodic costs of IPD and PNE by age and risk profile were estimated using data only from the MarketScan Database since it was the only source for which we had access to detailed patient-level data. Costs of IPD and PNE (per 100,000 person-years) by age and risk profile—as well as corresponding differences and 95 % CIs between risk groups—were estimated by combining sampled rates of disease (from all three data sources) and sampled age- and risk-specific unit costs (from MarketScan) via techniques of non-parametric bootstrapping (percentile method, 1000 replications with replacement). All analyses were conducted using SAS® 9.3 for Windows® (SAS Institute Inc., Cary, NC, USA).

## Results

### Risk profiles

Children (aged 0–17 years) who qualified for inclusion in the study contributed a total of 26.5 million person-years of observation (Table 1). Approximately 92 % of children had none of the selected chronic or immunocompromising conditions, 8 % had ≥1 at-risk condition (and no high-risk conditions), and <1 % had a high-risk condition. Among children with at-risk conditions, asthma (63 %), prematurity/low birth weight (13 %), neuromuscular/seizure disorders (10 %), and chronic lung disease (10 %) were the most common. Of children with ≥1 at-risk condition, 7 % had ≥2 such conditions.

**Table 1:** Rates of invasive pneumococcal disease and all-cause pneumonia among healthy, at risk, and high-risk persons

				Invasive Pneumococcal Disease						All cause pneumonia					
	No. of person-years			Age <18 years		Age 18–64 years		Age ≥65 years		Age <18 years		Age 18–64 years		Age ≥65 years	
	Age <18 years	Age 18–64 years	Age ≥65 years	Rate per 100 K	Rate ratios^a^ (95 % CI)	Rate per 100 K	Rate ratios^a^ (95 % CI)	Rate per 100 K	Rate ratios^a^ (95 % CI)	Rate per 100 K	Rate ratios^a^ (95 % CI)	Rate per 100 K	Rate ratios^a^ (95 % CI)	Rate per 100 K	Rate ratios^a^ (95 % CI)
Risk Group															
Healthy	24,406,759	63,445,448	5,389,930	2.5	--	2.7	--	8.3	--	587	--	458	--	1874	--
At-Risk	2,002,374	13,368,935	4,579,505	5.9	2.4 (2.0–2.9)	9.3	3.4 (3.2–3.7)	23.0	2.8 (2.5–3.1)	1801	3.1 (3.0–3.1)	1652	3.6 (3.6–3.6)	5662	3.0 (3.0–3.0)
Alcoholism	4591	333,634	23,905	0.0	--	20.4	7.5 (5.9–9.6)	41.8	5.0 (2.7–9.4)	501	0.9 (0.6–1.3)	2109	4.6 (4.5–4.7)	7400	3.9 (3.8–4.1)
Asthma	1,252,480	2,185,510	362,183	3.7	1.5 (1.1–2.0)	9.6	3.6 (3.1–4.1)	34.2	4.1 (3.4–5.0)	1718	2.9 (2.9–3.0)	2078	4.5 (4.5–4.6)	8570	4.6 (4.5–4.6)
Chronic heart disease	136,206	3,082,998	2,363,798	13.9	5.7 (3.6–8.9)	11.5	4.3 (3.8–4.8)	26.6	3.2 (2.8–3.6)	3699	6.3 (6.1–6.5)	2533	5.5 (5.5–5.6)	7100	3.8 (3.8–3.8)
Chronic liver disease	3759	292,697	50,540	26.6	10.8 (1.5–76.7)	24.6	9.1 (7.2–11.5)	53.4	6.4 (4.4–9.5)	3910	6.7 (5.7–7.8)	3002	6.6 (6.4–6.7)	7742	4.1 (4.0–4.3)
Chronic lung disease	192,731	1,251,143	882,061	8.3	3.4 (2.0–5.5)	27.0	10.0 (8.9–11.2)	51.1	6.2 (5.4–7.0)	3488	5.9 (5.8–6.1)	4802	10.5 (10.4–10.6)	12,379	6.6 (6.6–6.7)
Chronic use of oral steroids	22,508	275,267	65,775	13.3	5.4 (1.7–16.8)	8.0	3.0 (1.9–4.5)	15.2	1.8 (1.0–3.4)	1080	1.8 (1.6–2.1)	1164	2.5 (2.5–2.6)	3696	2.0 (1.9–2.1)
Diabetes	88,848	5,721,158	2,267,133	5.6	2.3 (0.9–5.5)	9.5	3.5 (3.2–3.9)	21.1	2.5 (2.2–2.9)	808	1.4 (1.3–1.5)	1683	3.7 (3.6–3.7)	5266	2.8 (2.8–2.8)
Down’s syndrome	20,657	7649	63	19.4	7.9 (2.9–21.0)	13.1	4.8 (0.7–34.4)	0.0	--	5514	9.4 (8.9–10.0)	2915	6.4 (5.6–7.3)	6316	3.4 (1.3–9.0)
Neuromuscular/seizure disorders	193,657	507,134	104,864	11.9	4.8 (3.2–7.3)	12.8	4.7 (3.7–6.1)	38.1	4.6 (3.3–6.3)	2536	4.3 (4.2–4.4)	2254	4.9 (4.8–5.0)	8539	4.6 (4.5–4.7)
Short gestation/low birthweight	249,576	34,580	90	12.0	4.9 (3.4–7.0)	5.8	2.1 (0.5–8.6)	0.0	--	2768	4.7 (4.6–4.8)	581	1.3 (1.1–1.5)	8864	4.7 (2.4–9.5)
Rheumatoid arthritis/Crohn’s/Lupus	--	579,373	162,206	--	--	17.8	6.6 (5.4–8.0)	33.3	4.0 (3.0–5.3)	--	--	2131	4.7 (4.6–4.7)	6465	3.5 (3.4–3.5)
Smokers	5019	2,128,945	180,504	0.0	--	12.5	4.6 (4.1–5.3)	34.9	4.2 (3.2–5.5)	777	1.3 (1.0–1.8)	1858	4.1 (4.0–4.1)	6691	3.6 (3.5–3.6)
High-Risk	119,692	3,062,400	1,774,181	48.5	19.6 (15.0–25.7)	26.3	9.7 (9.0–10.6)	36.7	4.4 (3.9–5.0)	3757	6.4 (6.2–6.6)	3094	6.8 (6.7–6.8)	7594	4.1 (4.0–4.1)
Chronic renal failure	17,112	356,088	344,160	64.3	26.1 (14.4–47.3)	47.2	17.5 (14.9–20.5)	50.0	6.0 (5.0–7.2)	4202	7.2 (6.7–7.7)	5566	12.2 (12.0–12.3)	11,873	6.3 (6.3–6.4)
Cochlear implant	1989	2517	1144	50.3	20.4 (2.9–145.0)	0.0	--	87.4	10.5 (1.5–74.8)	2062	3.5 (2.6–4.8)	1708	3.7 (2.8–5.0)	4544	2.4 (1.8–3.2)
Congenital immunodeficiency	21,047	76,771	14,392	42.8	17.3 (9.0–33.5)	87.3	32.3 (25.3–41.2)	118.1	14.2 (8.8–23.1)	4409	7.5 (7.0–8.0)	5919	12.9 (12.6–13.3)	14,738	7.9 (7.5–8.2)
Diseases of white blood cells	15,493	149,802	46,869	122.6	49.7 (31.5–78.5)	62.1	23.0 (18.6–28.3)	110.9	13.3 (10.0–17.8)	6048	10.3 (9.7–11.0)	6797	14.8 (14.6–15.1)	13,262	7.1 (6.9–7.3)
Functional/anatomic asplenia	19,321	109,298	42,976	119.0	48.3 (31.8–73.2)	93.3	34.5 (28.3–42.2)	116.3	14.0 (10.5–18.7)	8566	14.6 (13.9–15.3)	8721	19.0 (18.7–19.4)	15,976	8.5 (8.3–8.7)
HIV	1484	193,184	7306	0.0	--	46.6	17.2 (13.9–21.3)	27.4	3.3 (0.8–13.2)	2291	3.9 (2.8–5.5)	2457	5.4 (5.2–5.5)	6461	3.4 (3.2–3.8)
Immuno. conditions/drugs	63,702	2,495,776	1,523,021	54.9	22.3 (15.8–31.3)	24.4	9.0 (8.2–9.9)	36.4	4.4 (3.9–5.0)	3595	6.1 (5.9–6.4)	3146	6.9 (6.8–6.9)	7248	3.9 (3.8–3.9)

^a^Relative to healthy counterparts

Adults aged 18–64 years and ≥65 years contributed 79.9 million and 11.4 million person-years of observation, respectively. Approximately 79 % of adults aged 18–64 years had none of the selected chronic or immunocompromising conditions, while 17 % had ≥1 at-risk condition (and no high-risk conditions) and 4 % had a high-risk condition. The most common conditions in this age group were diabetes (43 %), chronic heart disease (23 %), and asthma (16 %); 18 % had ≥2 of any of the listed at-risk conditions. The prevalence of at-risk and high-risk conditions in persons aged ≥65 years was 39 % and 15 %, respectively; the most common conditions were chronic heart disease (52 %), diabetes (50 %), and chronic lung disease (19 %); 32 % had ≥2 such conditions.

### Clinical and economic burden

Rates of IPD and PNE were consistently higher among at-risk persons of all ages (IRR = 4.1 [95 % CI 3.9–4.3] and 4.5 [4.49–4.53]) and high-risk persons of all ages (IRR = 10.3 [9.7–11.0] and 8.2 [8.2–8.3]) versus their healthy counterparts. Rates were notably high for at-risk persons with ≥2 at-risk conditions (IRR = 9.0 [8.4–9.7] and 10.3 [10.3–10.4]), as well as those with asthma (IRR = 3.4 [3.0–3.8] and 4.5 [4.47–4.53]) or diabetes (IRR = 4.3 [4.0–4.6] and 4.7 [4.6–4.7]).

**Fig. 1:**
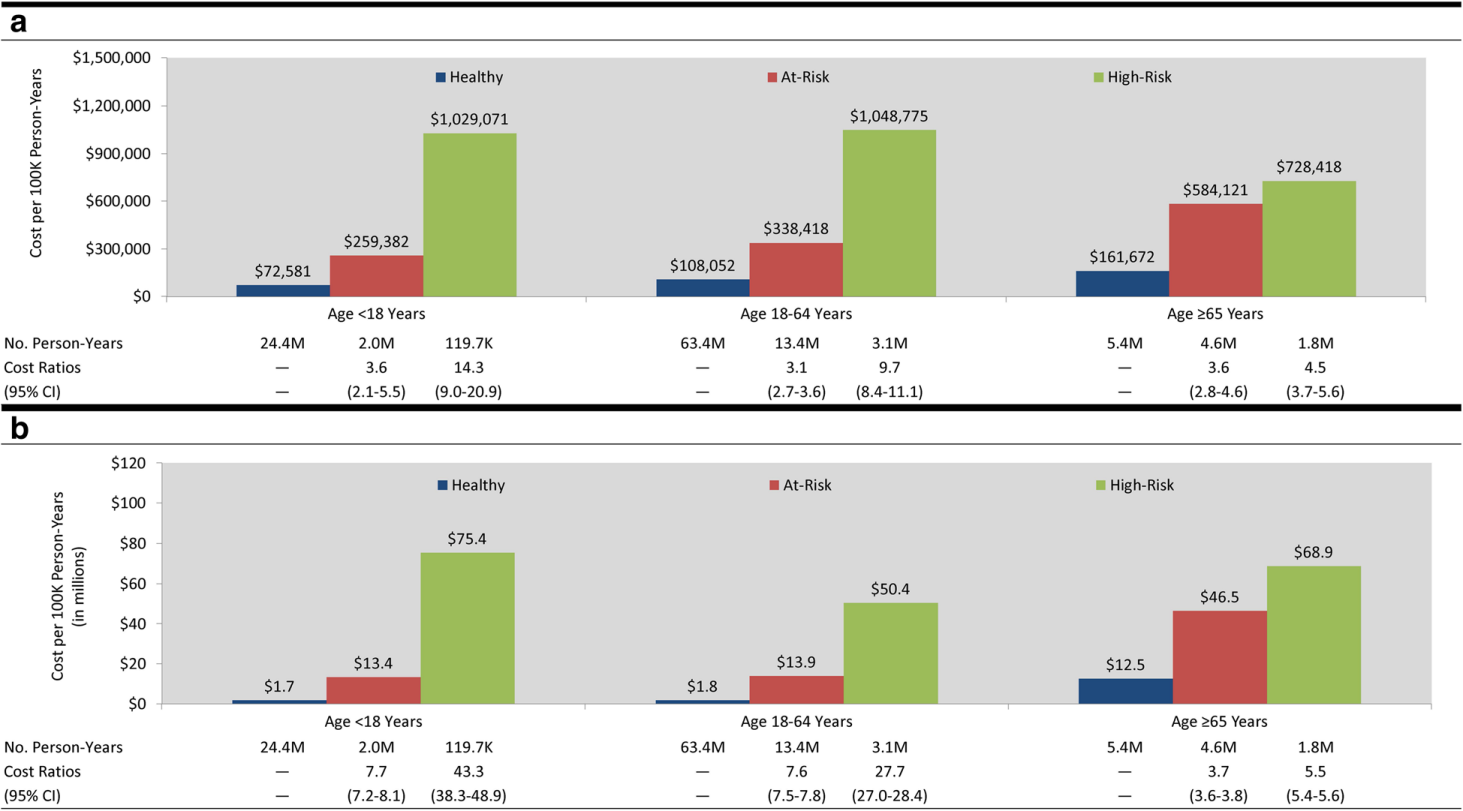
**a** Annual cost of invasive pneumococcal disease per 100,000 healthy, at-risk, and high-risk persons. **b** Annual cost of all-cause pneumonia per 100,000 healthy, at-risk, and high-risk persons

**Fig. 2:**
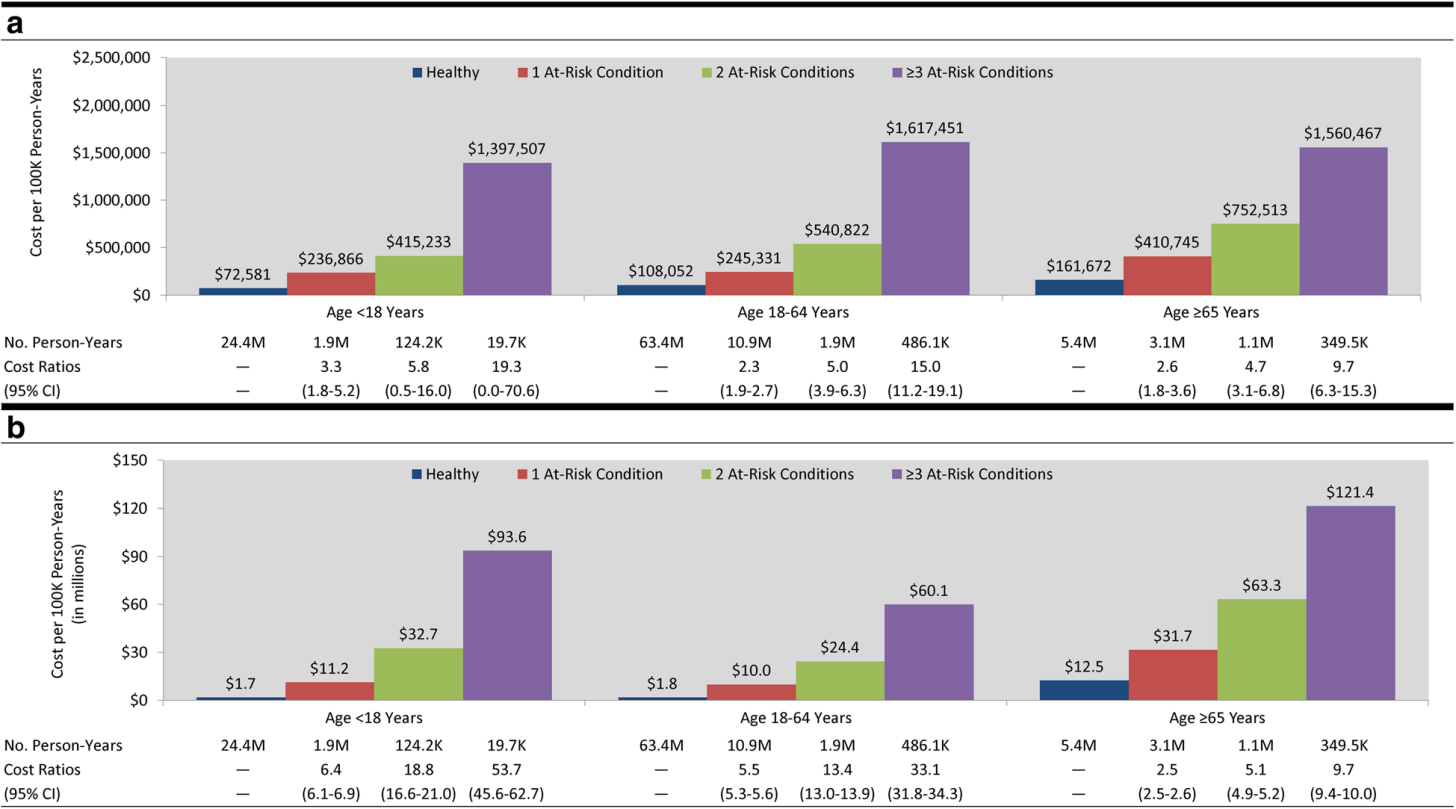
**a** Annual cost of invasive pneumococcal disease per 100,000 persons with at-risk conditions – by number of conditions – versus their healthy counterparts. **b** Annual cost of all-cause pneumonia per 100,000 persons with at-risk condition – by number of conditions – versus their healthy counterparts

Cost per IPD episode was generally similar across risk groups, while the cost per PNE episode was highest for the high-risk population followed by the at-risk and healthy populations (Table 2). Differences in economic costs of IPD and PNE per 100,000 person-years, by risk profile and number of at-risk conditions, were roughly the same as those for disease rates (Figs. 1 and 2).

**Table 2:** Costs and resource use per episode of invasive pneumococcal disease and all-cause pneumonia among healthy, at risk, and high-risk persons

	Invasive pneumococcal disease									All-cause pneumonia								
	<18 years			18–64 years			≥65 years			<18 years			18–64 years			≥65 years		
	INP	OP	Overall	INP	OP	Overall	INP	OP	Overall	INP	OP	Overall	INP	OP	Overall	INP	OP	Overall
Risk Group																		
Healthy																		
Cost																		
Mean	$50,744	$448	$32,879	$51,695	$499	$43,349	$22,733	$605	$20,897	$12,130	$484	$3280	$18,224	$592	$4247	$12,587	$487	$7015
SD	$82,620	$1540	$70,509	$84,974	$1148	$80,000	$28,405	$963	$27,876	$50,885	$717	$25,431	$37,336	$1043	$18,463	$26,113	$1141	$20,127
LOS (mean)	11.0	--	--	12.2	--	--	9.7	--	--	7.6	--	--	12.8	--	--	11.4	--	--
No. OP Visits (mean)	--	1.1	--	--	1.8	--	--	2.8	--	--	1.2	--	--	1.4	--	--	1.8	--
n	118	65	183	498	97	595	199	18	217	11,123	35,206	46,329	16,633	63,609	80,242	13,898	11,848	25,746
At-Risk																		
Cost																		
Mean	$101,491	$500	$47,111	$46,000	$1531	$39,377	$29,781	$412	$27,472	$19,207	$550	$7659	$21,815	$599	$8789	$13,719	$503	$8472
SD	$147,671	$927	$110,596	$64,018	$4362	$61,160	$93,161	$662	$89,765	$46,187	$792	$29,922	$46,562	$1073	$30,729	$26,672	$1461	$21,716
LOS (mean)	17.3	--	--	11.1	--	--	10.7	--	--	12.7	--	--	17.0	--	--	14.8	--	--
No. OP Visits (mean)	--	1.2	--	--	2.3	--	--	2.3	--	--	1.2	--	--	1.4	--	--	2.0	--
n	12	14	26	360	63	423	457	39	496	4007	6508	10,515	18,583	29,554	48,137	36,714	24,178	60,892
High-Risk																		
Cost																		
Mean	$29,896	$128	$22,891	$45,406	$715	$42,985	$23,809	$1714	$21,674	$35,721	$612	$20,304	$31,233	$750	$16,775	$15,090	$571	$9495
SD	$23,936	$83	$24,476	$52,315	$656	$51,872	$30,280	$4196	$29,536	$101,049	$925	$77,632	$62,950	$2477	$48,142	$26,296	$1745	$21,820
LOS (mean)	8.6	--	--	12.5	--	--	9.8	--	--	18.5	--	--	23.4	--	--	16.7	--	--
No. OP Visits (mean)	--	1.0	--	--	3.0	--	--	4.0	--	--	1.3	--	--	1.6	--	--	2.3	--
n	13	4	17	227	13	240	271	29	300	525	411	936	8480	7651	16,131	17,809	11,165	28,974

*INP* inpatient, *OP* outpatient

**Fig. 3:**
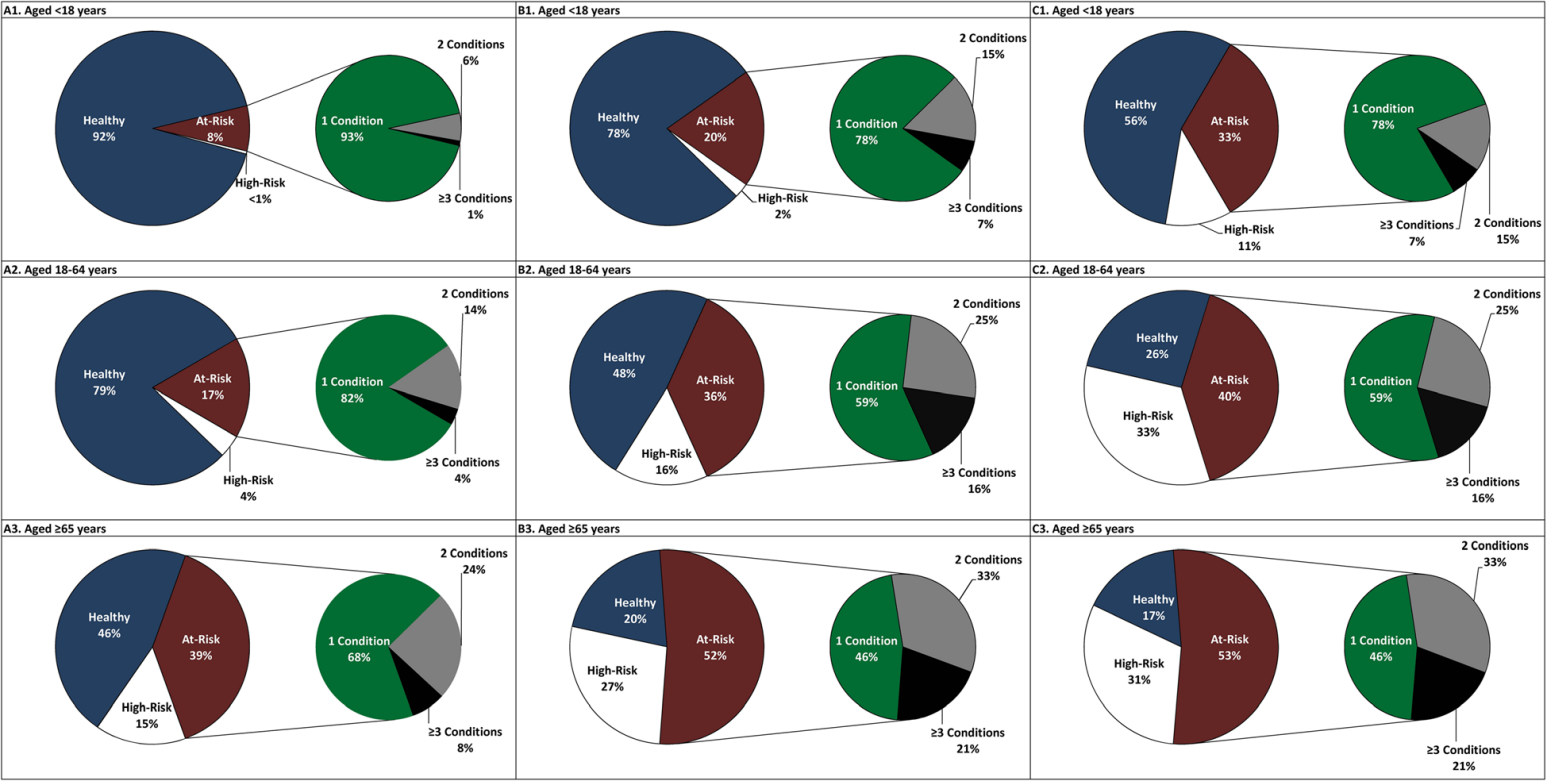
Distribution of persons by risk profile and among at-risk persons by number of conditions (Panel **a**), distribution of pneumococcal disease episodes among at-risk persons by number of conditions (Panel **b**), and distribution of pneumococcal disease costs among persons at-risk by number of conditions (Panel **c**)

Although high-risk persons were a small proportion of the total, they accounted for a substantial proportion of total costs (Fig. 3). On an overall basis, high-risk persons constituted only about 4 % of the population, but accounted for 18 % of cases of IPD/PNE and 30 % of the total cost of IPD/PNE. Similarly, persons with ≥2 at risk conditions, while constituting only 3 % of the total population, accounted for 21 % of total IPD/PNE costs. Economic costs increased in a graded fashion by severity of asthma (Additional file 1: Figure S1) and diabetes (Additional file 1: Figure S2).

## Discussion

Our results, based on analyses of data from three large repositories of healthcare claims, suggest that the increased rates of pneumococcal disease associated with certain comorbid conditions (i.e., among at-risk and high-risk persons) are closely aligned with increased healthcare costs in these populations. Elevated rates and higher episodic costs of IPD and PNE in at-risk and high-risk populations resulted in total healthcare costs that were 3–43 times higher (per 100,000 person-years)—depending on age and condition—compared with age-stratified healthy counterparts. Among adults aged 18–64 years, for example, the annual cost of PNE (per 100,000 persons) increased from $1.8 million for healthy persons, to $13.9 million for at-risk persons, and to $50.4 million for high-risk persons. Underlying the higher overall costs are increasing rates of disease (from 458, to 1652, to 3094 per 100,000 persons) and increasing mean cost per episode of disease (from $4247, to $8789, to $16,775). The increase in mean cost per episode is especially pronounced for cases of disease requiring inpatient care ($18,224, $21,815, and $31,233 across risk groups), which correlates with longer mean length of stay in hospital (12.8 days, 17.0 days, and 23.4 days, respectively). While a similar finding was expected for IPD, these results were not robust due to the relatively small number of identified episodes. Remarkably, while at-risk persons with ≥2 conditions and high-risk persons accounted for only 8 % of the population, they were responsible for more than 50 % of total IPD/PNE costs. In addition, among persons with at-risk conditions, elevated costs increased in a graded and monotonic fashion with the number of conditions present, and rates and costs of PNE were particularly high among persons with severe asthma and diabetes versus those with less severe disease.

The pattern of increasing costs across increasing levels of risk is similar to that observed in the above-cited prior study of the burden of pneumococcal disease among older US adults [2]. Although the risk categories considered in the two studies are similar, direct comparisons of costs with the prior study are challenging because they were estimated from different sources and were presented in a different manner. In the prior study, for example, direct costs of inpatient care were estimated on the basis of reported charges for the relevant conditions in the 2004 Healthcare Cost and Utilization Project Nationwide Inpatient Sample (HCUP NIS), after “stepping-down” costs to charges using hospital-specific cost-to charge ratios. In the current study, inpatient costs were estimated from paid amounts available in healthcare claims. In the prior study, costs were presented for the entire US population of older adults (i.e., those aged ≥50 years) stratified into four age groups, while in the current study costs were presented per 100,000 adults aged 19–64 years and ≥65 years, respectively.

Two additional findings merit further discussion. First, rate and cost ratios for IPD and PNE were similar. This finding likely is explained by two factors. The predominant manifestation of IPD is bacteremic pneumonia–69 % of all cases in the US in 2012—and pneumococcus is believed to be the leading bacterial cause of all-cause pneumonia [8, 10]. Thus, to a considerable extent, these two outcomes overlap and represent the same clinical manifestation—pneumococcal pneumonia. In addition, conditions that predispose to pneumonia caused by *S. pneumoniae* may increase the risk of disease due to other pathogens. For example, chronic lung disease probably increases the risk of pneumonia caused by influenza virus. The second finding was that rate ratios were smaller in older persons. We believe this finding to be a manifestation of the general decrease in immunocompetence that occurs with advancing age and that almost certainly diminishes the risk gradient between “healthy” older adults and those with established chronic medical conditions.

While healthcare claims databases provide information on large numbers of patients with specific diagnoses and the associated healthcare utilization, several limitations from the use of such databases for our study should be noted. First, use of operational algorithms and healthcare claims data resulted in an underestimation of the incidence of IPD (e.g., ~8 per 100,000 children <5 years of age) compared with national estimates from the Centers for Disease Control and Prevention (e.g., ~20 per 100,000 children <5 years of age) [10]. For IPD, it is likely that sometimes only the ICD-9-CM diagnosis code for unspecified septicemia or bacteremia is recorded on the healthcare claim form, without an accompanying diagnosis code for pneumococcus. Thus, while such encounters would be correctly identified as invasive disease, they would not be correctly identified as IPD. In addition, private healthcare claims databases—like the ones used in this study—include information for commercially insured populations, which often have an overall lower risk of disease. We note, however, that the age distribution of IPD cases in our study is similar to the distribution that has been reported by the Centers for Disease Control and Prevention (CDC). While it is not possible to formally evaluate the accuracy of our case-ascertainment algorithms within the context of this study, we did evaluate the sensitivity of our study results by employing alternative approaches to characterizing risk profiles and individual conditions. In one approach, relevant data available during the calendar year were considered as evidence that such conditions were present at the beginning of the calendar year, while in another approach, such evidence was treated as a change in risk status during the calendar year. Analyses also were conducted using less restrictive (i.e., more sensitive) algorithms in which any diagnostic, procedure, or drug information was considered evidence that such conditions were present. In addition, analyses were conducted by individual calendar year, with varying durations of look-back to ascertain risk profiles (e.g., ≤1 year for 2007, ≤4 years for 2010). Across all of the above-described analyses, our findings were largely unchanged.

Second, persons with public or no health insurance are not represented in the study databases, potentially limiting the generalizability of study results to other populations. Third, the costs of PNE are not a substitute for the costs of pneumococcal pneumonia, and thus our study undoubtedly overestimated the absolute clinical and economic burden of pneumococcal disease. For this reason, our study focused on rate ratios and cost ratios, rather the rates and costs per se. Assuming that the costs of pneumococcal pneumonia are similar to the costs of pneumonia due to infection with other pathogens, the costs of pneumococcal pneumonia would be proportional to the percentage of all-cause pneumonia due to *S. pne*umoniae, recently estimated for community-acquired pneumonia at 27 % (95%CI 21–29) [8]. Fourth, this study employed a retrospective cohort design and data from three large integrated healthcare claims databases to increase the precision of estimates. Although the three study databases comprise information from unique health plans and thus do not overlap at the plan level, it is possible that claims information from different time periods for the same patient may appear in each extract (and cannot be linked because of unique encrypted IDs). We believe such occurrences to be rare and the potential bias—if any—to be minimal. Finally, we did not include data on pneumococcal vaccination status in our analyses due to the lack of reliable information on this subject.

Several generic limitations of retrospective studies based on healthcare claims data also should be noted. All healthcare claims databases contain errors of omission and commission in coding, which undoubtedly results in some misclassification of study outcomes. Certain biases in coding may exist such that patients who, for example, are hospitalized for IPD or PNE may be more likely to have selected underlying at-risk/high-risk conditions listed as primary/secondary diagnoses on their claims, all else equal. Moreover, information often is not available for one or more clinically important parameters (in our study, for example, blood glucose/glycated hemoglobin [HbA1c] for diabetes, ejection fraction/New York Heart Association [NYHA] Class for heart failure), and pertinent medical history can be left-censored (e.g., diagnoses recorded before the time period of the study database are unobservable). The impact of these limitations on our results cannot be assessed within the scope of this study.

## Conclusion

In conclusion, the results of this study demonstrate that rates and costs of pneumococcal disease remain disproportionately high in persons with at-risk and high-risk conditions in the current era of widespread pneumococcal vaccination. Study results also demonstrate that rates and costs for individuals with ≥2 at-risk conditions approached those among persons with high-risk conditions, and that the rates and costs for persons with asthma and diabetes were especially increased.

### Ethics

Because the study databases were de-identified prior to their release to study investigators, their use for health services research is fully compliant with the HIPAA Privacy Rule and US federal guidance on Public Welfare and the Protection of Human Subjects. Formal IRB approval was therefore not required.

### Availability of data and materials

Use of the data for analyses described herein is restricted via license agreements between study investigators and the data vendors, and thus the data are not publically available. Use of the data sources for future research may be obtained via user-specific license agreements with the data vendors.

## Additional file

Additional file 1: Supplemental Material-Operational Algorithms and Additional Results. (DOCX 2446 kb)

## Abbreviations

ACIP: Advisory Committee on Immunization Practices
CDC: Centers for Disease Control and Prevention
HbA1c: glycated hemoglobin
HCPCS: Healthcare Common Procedure Coding System
HCUP NIS: Healthcare Cost and Utilization Project Nationwide Inpatient Sample
HIPAA: Health Insurance Portability and Accountability Act
ICD-9-CM: International Classification of Diseases, Ninth Revision, Clinical Modification
IMS: Intercontinental Marketing Services’
IPD: invasive pneumococcal disease
IRR: incidence rate ratio
NDC: National Drug Code
NYHA: New York Heart Association
PNE: pneumonia
US: United States
