# Supplementary material for: Rates and costs of invasive pneumococcal disease and pneumonia in persons with underlying medical conditions
Source: BMC Health Serv Res. 2016 May 13;16:182. doi: 10.1186/s12913-016-1432-4 (PMC4867996; doi:10.1186/s12913-016-1432-4)
Supplement: Additional file 1: — Supplemental Material-Operational Algorithms and Additional Results. (DOCX 2446 kb) [file 12913_2016_1432_MOESM1_ESM.docx]

**Online Supplement: Description of Data Sources**

Data for this study were collected from three large integrated healthcare claims databases during the period January 1, 2006 to December 31, 2010. The three databases—(1) Truven Health Analytics MarketScan® Commercial Claims and Encounters and Medicare Supplemental and Coordination of Benefits Databases; (2) IMS LifeLink™ PharMetrics Health Plan Claims Database; and (3) Optum Research Database—each comprise medical (i.e., facility and professional service) and outpatient pharmacy claims from a large number of private US health plans.

The MarketScan Database includes healthcare claims information from more than 50 employer-sponsored plans that provide health benefits via fee-for-service and fully/partially capitated products to over 15 million persons annually, including active/retired employees as well as their spouses and dependents. Plan members are geographically diverse (Midwest, 27%; Northeast, 12%; South, 41%; West, 20%), and approximately 21% are <18 years of age. The LifeLink Database includes healthcare claims information from over 75 US private health plans providing healthcare coverage via fee-for-service and fully/partially capitated products to approximately 15 million persons annually throughout the US (Midwest, 30%; Northeast, 14%; South, 36%; West, 20%)**;** ~24% of plan members are <18 years of age. The DataMart Database includes information from a large health plan that provides health benefits via alternative products (i.e., fee-for service, capitated) to over 7 million geographically-diverse persons annually (Midwest, 27%; Northeast, 11%; South, 47%; West, 16%); approximately 25% of plan members are <18 years of age. Thus, collectively, the three databases comprise claims information from health plans covering 37 million persons annually and, for this study, provide healthcare claims for a total of 129 million person-years of observation.

Data available from each facility and professional-service claim include dates and places of service, diagnoses (ICD-9-CM), procedures performed/services rendered (ICD-9-CM, HCPCS)**,** and quantity of services (professional-service claims)**.** Data available for each outpatient pharmacy claim include the drug dispensed, dispensing date, quantity dispensed, and number of days supplied. Selected demographic and eligibility information (including age/year of birth, sex, geographic region of residence, dates of plan eligibility) also is available.

Patient-identifying information was encrypted or removed from the study databases prior to their release to study investigators. Use of the study databases for health-services research is therefore fully compliant with the HIPAA Privacy Rule and federal guidance on Public Welfare and the Protection of Human Subjects (Public Welfare—Protection of Human Subjects; 45CFR 46 §46.101).
